# The Feasibility and Acceptability of Resilience Coaching for Adolescent Chronic Musculoskeletal Pain: A Single-Arm Pilot Trial

**DOI:** 10.3390/children9101432

**Published:** 2022-09-21

**Authors:** Sabrina Gmuca, Pamela F. Weiss, Mackenzie McGill, Rui Xiao, Michaela Ward, Maria Nelson, David D. Sherry, Peter F. Cronholm, Jeffrey S. Gerber, Tonya M. Palermo, Jami F. Young, Abby R. Rosenberg

**Affiliations:** 1 Department of Pediatrics, Division of Rheumatology, Children’s Hospital of Philadelphia, Roberts Center for Pediatric Research, 2716 South Street, 11-121, Philadelphia, PA 19146, USA; 2 Center for Pediatric Clinical Effectiveness, Children’s Hospital of Philadelphia, Philadelphia, PA 19146, USA; 3 PolicyLab, Children’s Hospital of Philadelphia, Philadelphia, PA 19146, USA; 4 Perelman School of Medicine, University of Pennsylvania, Philadelphia, PA 19104, USA; 5 Palliative Care and Resilience Program, Seattle Children’s Research Institute, Seattle, WA 98145, USA; 6 Department of Biostatistics, Epidemiology and Informatics, Perelman School of Medicine, University of Pennsylvania, Philadelphia, PA 19104, USA; 7 Mixed Methods Research Lab, Perelman School of Medicine, University of Pennsylvania, Philadelphia, PA 19104, USA; 8 Department of Family Medicine and Community Health, Center for Public Health Initiatives, Leonard Davis Institute of Health Economics, University of Pennsylvania, Philadelphia, PA 19104, USA; 9 Department of Pediatrics, Division of Infectious Diseases, Children’s Hospital of Philadelphia, Philadelphia, PA 19146, USA; 10 Department of Anesthesiology and Pain Medicine, University of Washington School of Medicine, Seattle, WA 98195, USA; 11 Center for Child Health, Behavior and Development, Seattle Children’s Research Institute, Seattle, WA 98145, USA; 12 Department of Child and Adolescent Psychiatry and Behavioral Services, Children’s Hospital of Philadelphia, Philadelphia, PA 19146, USA; 13 Cambia Palliative Care Center of Excellence, University of Washington, Seattle, WA 98104, USA; 14 Department of Pediatrics, Division of Hematology/Oncology, University of Washington School of Medicine, Seattle, WA 98195, USA

**Keywords:** pediatric rheumatology, resilience, adolescence, chronic musculoskeletal pain

## Abstract

Promoting Resilience in Stress Management (PRISM) is a well-established resilience coaching program for youth with chronic illness. It is a one-on-one intervention targeting skills in stress management, goal-setting, cognitive reframing, and meaning-making. We aimed to (i) assess the feasibility and acceptability of PRISM and (ii) explore PRISM’s impact on clinical outcomes among youth with chronic musculoskeletal pain (CMP). This was a single-arm pilot trial of PRISM for youth with CMP aged 12–17 years. Patients completed patient-reported outcome measures (PROs) pre- and post- intervention; patients and caregivers provided qualitative feedback. Twenty-seven patients were enrolled (63% enrollment rate); 82% percent were female. The patients’ median age was 16 years (IQR: 13–16). The intervention completion rate was 81% (*n* = 22). The mean satisfaction for PRISM overall was 4.3 (SD 0.9), while the mean acceptability of the intervention measure (AIM) was 4.4 (SD 0.89). Participants reported improved resilience (2.2 [SD 5.1]), functional disability (−3.5 [IQR: −6.0, 1.0]), and psychological distress (−1.0 [−5.0, 2.0]) from baseline to immediately post-treatment; pain intensity, pain catastrophizing, and global health were similar at both time points. Feedback was positive and suggested that a group component may be helpful. PRISM is feasible and acceptable among youth with CMP. Exploratory analyses suggest improvements in clinically relevant outcomes, warranting further investigation.

## 1. Introduction

Evidence-based treatment for adolescent chronic musculoskeletal pain (CMP) is rooted in a biopsychosocial framework and a multidisciplinary context [1], including a combination of rehabilitative therapies [2,3] and cognitive behavioral therapy (CBT) [4]. However, the uptake of evidence-based non-pharmacological interdisciplinary treatment into regular use by practitioners is suboptimal [1]. With an estimated 3 million annual visits [1] and annual costs of up to USD 19.5 billion for adolescent CMP, successful treatment of high-impact chronic pain is critical [5]. Increased accessibility to alternative, cost-effective, psychosocial interventions that are acceptable to youth with CMP and can be delivered as part of usual care are needed in order to increase treatment engagement and improve care.

Resilience coaching may serve as an alternative, adjunctive intervention for managing adolescent CMP. Promoting Resilience in Stress Management (PRISM) is a resilience coaching program developed for youth with serious illness [6]. PRISM has excellent feasibility and acceptability in adolescent chronic illnesses such as cancer, type 1 diabetes, and cystic fibrosis [6,7], as well as efficacy among adolescents with cancer [8,9]. PRISM is a one-on-one, brief, and remotely deliverable skills-based intervention targeting four reproducible “resilience resources” (i.e., stress management, goal-setting, cognitive reframing, and meaning-making) [6]. PRISM sessions are led by trained, bachelor’s-degree-level, non-clinical staff (rather than licensed practitioners, who are already in overly high demand). In doing so, PRISM alleviates system burdens and delivers care that otherwise may be difficult to access. Because it is a brief, manualized intervention, PRISM can be widely disseminated with excellent fidelity [10]. This, combined with its capability to be delivered remotely, makes it highly appealing in routine clinical care.

In a previous cross-sectional cohort study, we found that the mean level of patient-reported resilience according to the validated Connor–Davidson Resilience Scale (CD-RISC-10) was low in youth with CMP, and lower levels of self-perceived resilience were associated with poorer health-related quality of life, greater functional disability, and greater pain [11]. We hypothesized that PRISM teaches skills that improve pain coping, thereby resulting in improvements in pain-related clinical outcomes (including functional disability and psychological distress). Therefore, the main objectives of this study were to determine the feasibility and acceptability of PRISM among youth with CMP. Additional objectives included to (1) determine the preferred mode of delivery of PRISM; (2) explore PRISM’s impact on health-related quality of life, resilience, functional disability, psychological distress, pain catastrophizing, and pain intensity; and (3) identify any potential adaptations of PRISM for CMP.

## 2. Materials and Methods

Study Design: This study was a single-center, prospective, feasibility and acceptability pilot trial using a mixed-methods approach (quantitative survey data and sequential explanatory, semi-structured interviews).

Setting and Participants: From 9/2020 to 4/2021, we enrolled English-speaking patients aged 12–17 years, diagnosed with chronic (≥3 months in duration) musculoskeletal pain, and seen for an initial evaluation in an outpatient pediatric rheumatology non-pharmacological interdisciplinary pain clinic, along with one of their caregivers (i.e., legal guardians). We excluded youth with cognitive impairment precluding the completion of survey measures or PRISM exercises, those without a legal guardian providing consent for participation, and youth with inflammatory, neurological, and/or oncological/hematological diagnoses.

Potential study subjects were approached via telephone/e-mail after their initial clinic visit. Patients provided informed consent to complete surveys and participate in the intervention and semi-structured interviews. Caregivers consented to participate in session 5 of PRISM and the semi-structured interviews. The study received institutional review board approval from the Children’s Hospital of Philadelphia. Child participants received 25 USD/survey and 25 USD/interview. Parent participants received 20 USD/visit for their time as well as 15 USD/interview.

Study Intervention: PRISM is a resilience coaching program targeting resilience and coping skills over the course of four 1:1, 30–50-min sessions, administered approximately 1–2 weeks apart (totaling about 3 months in duration), followed by a 30-minute optional 5th follow-up session (Table 1). The 1st session occurred within 2–4 weeks of enrollment. Patients chose the mode of delivery: in person, via HIPAA-compliant web-based communication, or by telephone.

PRISM is a manualized intervention. Coaches are required to have completed a bachelor’s-level degree. They undergo standardized training, including dynamic role-playing, to demonstrate competency (including an 8-hour training session and delivery of the intervention to 2 peers, with self-evaluation and peer evaluation at completion). Sessions 1–4 are required, and include stress management, goal-setting, cognitive reframing, and meaning-making (Table 1). The 5th and final session involves an optional, coach-facilitated family meeting.

For this study, a separate team at Seattle Children’s Hospital (SCH) supported PRISM coach training and fidelity monitoring. PRISM sessions were audio recorded. The PI or supervising team member at SCH assessed the fidelity of the first 5 sessions for each coach, after which one of each 5 subsequent sessions was randomly selected to be monitored for fidelity, with feedback and retraining regarding adherence to the protocol, and the approach was refined as needed.

Data Collection: Demographic and clinical data were abstracted from the electronic health records and existing data in the clinic’s IRB-approved prospective patient registry. At baseline and immediately post-treatment, adolescents completed patient-reported outcome measures, and at post-treatment they also completed a survey regarding the acceptability of the intervention.

Patient-reported outcome measures were included in both pre- and post-treatment surveys. Health-related quality of life was measured by the *PROMIS Pediatric Global Health 7* (*PGH-7*) [12,13], where raw scores range from 7 to 35 and are translated to T-scores ranging from 16.0 to 67.5, with higher scores indicating greater global health. Self-perceived resilience was measured with the *10-item Connor–Davidson Resilience Scale* (*CD-RISC-10*) [14], where scores range from 0 to 40, with higher scores indicating greater self-perceived resilience. Functional disability was measured by the *Functional Disability Inventory* (*FDI*) [15], where scores range from 0 to 60, with higher scores indicating greater functional disability. Psychological distress was measured by the *Kessler-6 Psychological Distress Scale*, ranging from 0 to 24, with higher scores indicating greater nonspecific psychological distress [16]. Pain catastrophizing was measured with the P*ain Catastrophizing Scale, Child report* (*PCS-C*), where total scores range from 0 to 52, with higher scores indicating greater pain catastrophizing [17], while pain intensity—ranging from 0 to 10, with greater scores indicating greater pain—was evaluated by the *PROMIS Pediatric Numeric Rating Scale v1.0-Pain Intensity* [18].

Post-intervention surveys included measures on satisfaction and acceptability. Patients completed both overall and module-specific satisfaction scores, rated on a 5-point (1–5) Likert scale (ranging from very dissatisfied to very satisfied). They also completed the Acceptability of Intervention Measure (AIM) [19,20], where respondents rate their agreement with a few statements (1 = completely disagree; 5 = completely agree); the mean summary score indicates the level of acceptability. After completing PRISM, participants and their caregivers were invited to participate in audio-recorded semi-structured qualitative interviews.

## 3. Statistical Plan

Quantitative Data: The main endpoints for this study were feasibility and acceptability. Feasibility of the intervention’s delivery (binary) was defined as ≥70% of participants completing all four required sessions. Given the previously reported retention rates for PRISM of 80–90% among youth with cancer and type 1 diabetes, we hypothesized that our retention rate would be lower among youth with CMP due to less-frequent clinical care visits and, therefore, fewer opportunities to coordinate study visits with routine care [6,8]. We reported the number of eligible participants approached, the proportion of those participants enrolled in the study (enrollment rate), and the mean percentage of sessions (PRISM 1–4) attended among the enrolled participants. The goal enrollment rate was defined at ≥25% based on a prior enrollment rate of 58% among patients with type 1 diabetes. Since CMP is not life-threatening, we anticipated a lower likelihood of enrollment in our cohort. PRISM was defined as acceptable if the AIM score was ≥3 on a 5-point Likert scale and the mean satisfaction score for PRISM overall (i.e., the average over all sessions) for patients was ≥3 on a 5-point Likert scale. Secondary endpoints included the mean (or median, as appropriate) group change in patient-reported outcomes at study completion compared to study enrollment, as well as the preferred format of the PRISM intervention (i.e., the format comprising the greatest proportion of total visits 1–5).

Baseline and demographic characteristics were summarized by standard descriptive summaries. Change scores were calculated by subtracting baseline scores from follow-up scores and averaged; paired *t*-tests (for parametric variables) and Wilcoxon matched-pairs signed-rank tests (for non-parametric variables) were conducted to detect changes from baseline to follow-up; *p*-values < 0.05 were considered statistically significant.

Qualitative Data: Patients and caregivers were asked to share their perceptions of the PRISM program via semi-structured interviews. Interview guides were directed at the intervention content, timing, duration, and delivery. Study participants were approached for semi-structured interviews upon completion of session 5 or study exit—whichever occurred first. We aimed a priori to enroll a total of 20 evaluable dyads to reach saturation of qualitative themes. Audio recordings were transcribed by Datagain Services (Secaucus, NJ) and de-identified. Transcripts were then entered into NVivo 1.5 Plus (QSR International, Burlington, MA, USA) for coding and analysis. Two members of the study team coded the transcripts independently and met to identify emergent themes according to content analysis [21,22]. All discrepancies were explored by the complete study team and resolved through consensus.

## 4. Results

Participants: We approached 43 eligible patients and enrolled 27 (63%). Of the 16 eligible subjects who did not enroll, 3 (19%) reported time constraints, 3 (19%) were uninterested, and 10 (62%) did not reply to the invitation. Of the 23 enrolled subjects, 85% completed all four required PRISM sessions. Of the 23 participants who completed the intervention, 1 participant did not complete the post-intervention surveys, resulting in 22 evaluable subjects.

The median age of the participants was 15 years (IQR: 13, 16); most of them were female (82%), Caucasian/white (81%), and non-Hispanic (96%) (Table 2). At study entry, they reported pain symptoms for a median of 18 months (IQR: 12, 49). Their pain intensity was moderate (median 6.5 [IQR: 5, 8]) and, on average, the patients had moderate functional disability [23]. More than half had comorbid anxiety and/or depression. At the time of enrollment, 55% (*n* = 12) of participants reported receiving mental health services. Two participants (9%) received treatment in the hospital’s intensive interdisciplinary pain rehabilitation program during the study. Of the 27 enrolled subjects, the initial survey completion rate was 96%, and the final survey completion rate was 85%.

PRISM Feasibility: Feasibility metrics were achieved. Twenty-three (85%) of the enrolled participants completed all four core PRISM sessions (mean 3.7 sessions completed, SD 0.71); twenty-two (96% of the 23 who completed the four core sessions; 81.5% of all enrolled) attended the optional fifth session.

Acceptability: Acceptability metrics were also achieved. Twenty (91%) participants reported high satisfaction with PRISM overall (≥4 on a 5-point Likert scale) (Table 3). For individual items on the AIM, the mean scores were as follows: (1) PRISM meets my approval (4.4 [0.80]); (2) PRISM is appealing to me (4.2 [1.02]); I like PRISM (4.5 [0.96]); I welcome PRISM (4.5 [0.96]).

Of those who completed all four PRISM sessions (*n* = 23), 15 subjects (65%) used only video visits for all PRISM sessions, while 5 subjects (22%) solely utilized telephone visits. One participant completed all sessions as video visits except for one in-person session, which was coordinated with clinical care. One participant started with video visits but then switched to telephone visits due to poor internet connectivity. Only one participant chose in-person sessions for all PRISM sessions.

Qualitative interviews with patients and their caregivers (*n* = 22 dyads) lasted an average of 26 min (range 16–60 min). Interviewees expressed that PRISM’s logistical aspects worked well for them (Table 4). They felt that the timing of the sessions fit within their schedules, that the program and session lengths were appropriate, and that the frequency of sessions was sufficient. Interviewees were enthusiastic about PRISM being offered remotely. They also appreciated the coach’s flexibility in scheduling and projection of a casual environment for visits.

Most interviewees felt that PRISM provided a valuable set of tools to manage the psychological components of pain. Participants liked that the program focused on building a positive attitude, integrated psychological and physical treatment strategies, and helped them reduce their overall stress. Caregivers saw a clear connection between their child’s improved ability to manage their stress and their ability to manage pain.

Patients and caregivers wanted to participate because patients were struggling with CMP and wanted to learn coping strategies, to help others with CMP through the program’s research findings, or because they had difficulty finding psychological services outside of PRISM. Patients said that PRISM reinforced their existing belief that therapy is beneficial and reinforced their desire to continue working on their resilience in therapy, including potentially with a provider using CBT. Several mentioned thinking that PRISM would be useful as a “gateway” to therapy for other youth who might be new to or skeptical about therapy.

Suggestions for improvement included having caregivers attend sessions to ensure that they knew the material. Other suggestions included adding group sessions for the patients (or another social component to share skills and build community), providing printed workbooks to reinforce the session content, and offering a crisis hotline.

Exploratory patient-reported outcomes: Functional disability improved by −3.5 points (IQR: −6.0, 1.0; *p* = 0.01) (Table 5). Self-perceived resilience increased by 2.2 points (SD 5.1) post-intervention (*p* = 0.06). Psychological distress improved by 1 point [(IQR: −5.0, 2.0); *p* = 0.09]. Pain-catastrophizing (mean change = −3.5 [IQR: −14.0, 7.0]; *p* = 0.19), pain intensity (mean change = 0.0 [IQR: −1.0, 0.0]; *p* = 0.17), and HRQOL (mean change= 0.6 points [SD 4.4]; *p* = 0.52) were similar pre- and post-intervention.

## 5. Conclusions

In this study of PRISM for adolescent CMP, we demonstrated a high enrollment rate of 63%, an excellent retention rate of 85%, and high scores for general satisfaction and acceptability. This was complemented by positive themes of the helpfulness of PRISM from qualitative data. Furthermore, exploratory evaluation of changes in PROs suggested potential improvements in resilience and important clinical outcomes via resilience coaching.

The findings from this pilot study are consistent with those of prior trials of PRISM in which high patient satisfaction with PRISM and improvements in self-perceived resilience and quality of life, along with lower psychological distress, were found for youth with other chronic illnesses [6,8,25]. Additionally, among adolescents and young adults with cancer, PRISM improved patient-reported cancer-related quality of life and hope [26]. Taken together, these findings suggest that the resilience resources targeted by PRISM are critical and universal for improving stress management in the setting of adolescent chronic illness. What is unique to our study is the assessment of pain and pain-related measures as exploratory PROs. Our findings suggest that PRISM may target functional disability and that measures of functional disability may be important in future works assessing PRISM in pediatric chronic illnesses where pain is a major contributor to disease severity.

Patients in our study were able to choose the mode of delivery. They favored video visits, citing the convenience of this format. Patients did not endorse any negative impact that the virtual format may have had on building rapport with the PRISM coach. If anything, they noted that the location of the PRISM coach affected the tone and mood of the session, with patients preferring the coach being in a casual environment (e.g., on a couch rather than at an office desk). This contrasts with youth with cystic fibrosis who, in previous research, reported appreciating the convenience of PRISM’s delivery in the inpatient setting and the fact that sessions were more engaging in person rather than remotely [25]. Moreover, our study was conducted during the COVID-19 pandemic; patient preferences may have reflected changing norms or preferences in digital–clinical interactions. Taken together, while the skills in PRISM may be universal across pediatric chronic illnesses, the preferred delivery and setting for the intervention may differ based on disease type, severity, or evolving norms. PRISM’s ability to be delivered in several settings and modes allows for its generalizability across various patient populations.

One recommendation identified from the qualitative feedback in our study was the addition of a group component or group session to PRISM. Wakefield et al. [27] demonstrated that concealment is a common coping strategy employed by adolescents with chronic pain, generally leading to social isolation. We suspect that the addition of a group session to PRISM may be welcomed by youth with CMP and could mitigate social isolation. In fact, PRISM has been successfully restructured and delivered at the group level for healthcare workers during the COVID-19 pandemic; thus, this format could be relatively easily assessed among youth with CMP [28,29].

Limitations: There are limitations to our study. The first is that this study, without a control group, was not designed to assess the efficacy of the intervention. We were also not able to assess moderators of intervention effects; it is unclear whether all youth with CMP would obtain equal benefits from PRISM. Similarly, our sample size was too small to explore “dose” effects or the impact of individual sessions on patient perceptions and outcomes. Our qualitative data suggest that certain sessions were more resonant for some participants and that all four core sessions were perceived as valuable. It is important to highlight that 55% of youth in our sample were already in psychological treatment and, therefore, our cohort was relatively heterogeneous with regards to prior exposure to mental health services. Furthermore, patients may have been prescribed other mental health treatments—including pharmacological treatments—over the course of the study interval. Therefore, we cannot attribute changes in patient-reported outcome measures solely to the effects of PRISM. This study is lacking in generalizability, as we did not have diverse racial or ethnic representation, and this was a single-center study. Another limitation is that we provided flexibility in scheduling of PRISM sessions, with patients able to participate in sessions on weekdays between 8 a.m. and 6 p.m. This may not be readily feasible in real-world clinical settings, depending on staffing. Similarly, the one-on-one format of the intervention may be too labor- and time intensive for clinical practice, highlighting the value of further researching the delivery of PRISM in a group setting. Future multicenter studies will be sufficiently powered to address these limitations and guide future implementation efforts.

Clinical Implications for Pediatric Chronic Pain: This study has implications for routine clinical care in pediatrics. Many pediatric practices care for patients with CMP without access to the resources available in pediatric chronic pain clinics or intensive rehabilitation programs. This, compounded with long wait times for new patient visits with mental health providers, leaves youth with CMP vulnerable to worsening functional disability and psychological distress as they await appropriate care. Perceived stigma is a known barrier to youth establishing care with a mental health provider. Our qualitative findings suggest that some youth experience PRISM as a positive introduction to CBT-based skills, which may decrease stigma around engagement in further mental health treatment. Other youth were already engaged in CBT or other mental health services; for them, their prior engagement in mental health services may have served as a critical foundation for an interest in participating in PRISM, which then reinforced their confidence in and practice of positive psychological skills. Either way, PRISM may be an important adjunct service for this population. Additionally, the minimal training required to implement PRISM and the utilization of a telehealth format allow for relatively easy implementation of PRISM in otherwise resource-limited pediatric practices. Such incorporation of PRISM into routine clinical care would allow for the delivery of pain coping skills that may be adequate for some youth and negate their need for individual counseling, and for others may serve as a critical interim pain management skillset until formal cognitive behavioral therapy can be initiated. While PRISM would not serve as a complete panacea for clinic wait times, the downstream effects of implementation of PRISM into clinical care could include prioritization of the limited appointments in pediatric chronic pain clinics for those with the greatest need for multidisciplinary care. Formal assessment of the efficacy of PRISM in CMP, as well as other pediatric diseases impacted by chronic pain, is worthy of future investigation.

Summary: PRISM is a feasible and acceptable resilience coaching program for youth with CMP that is attractive due to its remote delivery and limited training requirements. Feedback was generally positive, with the suggestion of a group component as a possible adaptation. Formal examination of the efficacy of PRISM as well as mediators and moderators of intervention effects is needed to inform future implementation efforts and the incorporation of resilience coaching into routine clinical care across multiple settings.

## Figures and Tables

**Table 1:** PRISM sessions’ content and structure.

PRISM	Topic	Skills	Details	Format	Recipient
1	Managing stress	Mindfulness; relaxation	Mindfulness strategies including deep breathing techniques, relaxation strategies, mindfulness meditation, discussion of mindfulness versus mindlessness and overthinking, and acceptance via observing emotions without judgment and acknowledging them	Option of in person, telehealth, or telephone, and in accordance with hospital policies surrounding COVID-19	Patient only
2	Setting goals	Setting specific and realistic goals; planning for roadblocks	Setting specific, realistic, desirable goals, planning for roadblocks, strategies for dealing with roadblocks, and identifying how parents/caregivers can help meet goals		
3	Positive reframing	Recognizing and replacing negative self-talk	Recognizing negative self-talk, identifying unrealistic/negative thoughts, and replacing these thoughts with positive/manageable ones		
4	Making meaning	Identifying benefits, gratitude, purpose, and legacy	Reframing current experience into a meaningful one, self-reflection/mindfulness, journaling		
5	Coming together	Discussion of what worked	Discussion of strategies practiced, identification and recognition of successes, identification of further needs, referrals to additional resources, and shared conversation with parents: What works? How can family help?		Patient and proxy
Skill practice			Between-session exercises to practice, further develop, and track skills	Digital app and/or worksheets	Patient

**Table 2:** Pre-intervention demographics and clinical characteristics.

	Total N = 22
Age, median (IQR)	15 (13, 16)
Female, n (%)	18 (81.8%)
Non-Hispanic, n (%)	21 (95.5%)
Race, n (%)	
White	18 (81.2%)
Black	2 (9.1%)
Mixed race	1 (4.5%)
Asian	1 (4.5%)
Duration of symptoms (months), median (IQR)	18 (12, 48)
Pain distribution, n (%)	
Diffuse pain	16 (72.7%)
Localized pain	6 (27.3%)
Constant pain, n (%)	19 (86.4%)
Patient FDI (0–60), median (IQR)	29.5 (13, 37)
Parent FDI (0–60), median (IQR)	25.5 (13, 34)
Presence of allodynia, n (%)	17 (77.3%)
Anxiety, n (%)	14 (63.6%)
Depression, n (%)	12 (54.6%)
PROMIS pain intensity (0–10), median (IQR)	6.5 (5, 8)
Least pain (0–10), median (IQR)	3 (2,5)
Most pain (0–10), median (IQR)	10 (9,10)
PROMIS fatigue, n (%)	64.6 (16.8)
PROMIS peer relationships *	46.3 (12.8)

Legend: *, Missing for *n* = 2. Anxiety and depression were self-reported. PROMIS = Patient-Reported Outcomes Measurement Information System; scores reported as T-scores with a mean of 50 and SD of 10 in the reference population. All measures are self-reported other than parent-reported functional disability (FDI).

**Table 3:** Satisfaction with PRISM among youth with CMP (*n* = 22).

PRISM Session	Skills	Satisfaction Score, Mean (SD)
1: Managing stress	Mindfulness; relaxation	4.3 (0.99)
2: Setting goals	Setting specific and realistic goals; planning for roadblocks	4.1 (0.87)
3: Positive reframing	Recognizing and replacing negative self-talk	4.2 (1.01)
4: Making meaning	Identifying benefits, gratitude, purpose, and legacy	4.2 (0.85)
5: Coming together (optional)	Discussion of what worked; option to include caregivers	4.0 (1.2)
**Average Overall**		**4.3 (0.94)**

**Table 4:** Patient and caregiver feedback on PRISM.

Overall helpfulness and benefits	“I found it helpful as I wouldn’t say kind of counseling, but it definitely helped to remind myself and learn new skills about ways that I can help manage my stress and my pain and just overall coping with my condition. I found it extremely helpful, learning new things, and going over it with somebody, personally being able to talk about it and come up with personal examples.” (*Patient*) “In the beginning, when they came into the doctor’s office, I was like, what is this? And then actually going through that, I think help with, like, coping mechanisms, like, calm myself down or to relax myself, or to just focus on that, and not have to worry about, like, everything going on around me.” (*Patient*)
Decision to participate	“We never, ever, ever would have thought ourselves to be in this situation and I think anything that can help these kids, she and I are 100% for, because I know that if someone’s willing to do some research and help try and figure out what can help people with this condition because it’s awful, it’s terrible. So that’s what we’re for, we appreciate people caring enough to try and figure out a way another tool to help them.” (*Caregiver*) “Like it’s a gift card, really…I have a shopping addiction and it helps feed my shopping addiction, so anything to feed my shopping addiction....I didn’t have a therapist, and if this is the closest thing I could get to therapy, I would take it, because how I deal with stress … it’s probably like the normal response, but it’s not the healthy response to do, especially when you’re dealing with the amount of stress that I’m dealing with, and probably as an adult, you’re dealing with this normal amount of stress, but as a teenager seems like a lot more.” (*Patient*)
Session logistics	“I thought that it was nice to have it every week. And I feel like, if you had more than once a week, that might be a little too much. But how it was, it was good. And then, how long it lasted, I thought that it was like a good period of time, because it was a little over a month. So that was kind of good, because you got used to it.” (*Patient*)
Accessibility	“Because of [PRISM] being remote, that made it incredibly doable. I don’t think we would have participated, honestly, even though I wanted to, if we were driving up there because we lived in [Different State]. But because it’s being remote, that made it is completely acceptable and flexible and the amount of time, it was like, you’d either find the amount of time, whether it was half an hour, an hour and a half, it wouldn’t have mattered and we could just find it in our schedule.” (*Caregiver*)
Effect of PRISM on patients’ mental health perceptions	“I think it [PRISM] would help [others who have never worked with a psychologist] because in the sessions, I felt like very able to voice my opinions and just very heard and not judged. So, I think it would help.” (*Patient*) “I’ve always really [been] opened [to therapy] so I’ve had that relationships since I was really, really young. And I’m really glad to have people in my life who support things like that. But also [there are] people in my family that don’t support things like psychologists or psychiatrists who don’t believe in stuff like that. So, I think if there was a patient who was kind of afraid of that [therapy], this is a really nice way to open their mind to psychology and stuff like that…it’s much better than going from just AMPS to a psychiatrist…” (*Patient*)
Caregiver interest in resilience	“I would be [interested in resiliency coaching myself] because I feel like as my child is going through the pain that they are going through, it’s not easy, and it’d be great to have the tools to handle that, because I feel like as a caregiver, as a primary caregiver, you do need the resilience coaching as well, so.” (*Caregiver*) “Sure, possibly because it’s a lot of stress when your kid is in pain. I mean, I kind of feel like I said, I’ve been around for a while, I’m pretty good, I’m pretty resilient, just life makes you that way after a while, you know, so I don’t know if it’s something that I necessarily have to take part in…but I think that if I hadn’t had that [past group therapy program], you know, if my daughter hadn’t had that, like, yeah, absolutely, something that I would want to do kind of alongside her to say, ‘Alright, yeah, we’re learning how to deal with stress together.’” (*Caregiver*)

**Table 5:** Changes in patient-reported outcome measures pre- and post-treatment for youth with CMP (*N* = 22).

*Domain*	*Measure*	*Pre-Treatment*	*Post-Treatment*	*Change* (*Post–Pre*) *	*p-Value*	*Reference Ranges*
***Overall HRQOL***	**PROMIS Pediatric Global Health 7**	40.5 (13.6)	41.1 (12.6)	0.6 (4.4)	0.52	MCID = 3.0 T-score with a mean of 50 and SD of 10
***Resilience***	**10-Item Connor–Davidson Resilience Scale (CD-RISC 10) [0–40]**	25.0 (8.8)	27.2 (7.4)	2.2 (5.1)	0.06	MCID = 4.4 ^
***Functional disability***	**Functional Disability Inventory (FDI) [0–60]**	24.5 [8.0, 35.0]	16.5 [7.0, 31.0]	−3.5 [−6.0, 1.0]	0.01	MCID = 8.0 0–12: no/minimal 13–20: mild 21–29: moderate ≥30: severe
***Psychological distress***	**Psychological distress (K6 total score) [0–24]**	9.5 [4.0, 13.0]	5.5 [3.0, 11.0]	−1.0 [−5.0, 2.0]	0.09	MCID = 3.0 ^ ≥7 = high distress ≥13 = debilitating distress
***Pain catastrophizing***	**Pain catastrophizing (PCS-C Total) [0–52]**	14.5 [7.0, 31.0]	15.0 [2.0, 24.0]	−3.5 [−14.0, 7.0]	0.19	0–14: low 15–25: moderate ≥26: high
***Pain intensity***	**PROMIS pain intensity [0–10]**	6.5 [5.0, 8.0]	6.0 [5.0, 7.0]	0.0 [−1.0, 0.0]	0.17	MCID = 2.0 [24]

Legend: PRISM = Promoting Resilience in Stress Management. CMP = chronic musculoskeletal pain. MCID = Minimal clinically important difference. PROMIS PGH7 = Patient-Reported Outcomes. * The mean of subject level differences in scores post-intervention compared to pre-intervention. Measurement Information System pediatric global health 7 (T-score with a mean of 50 and SD of 10 in the reference population). K6 = Kessler-6 Psychological Distress Scale, with scores ≥7 consistent with high distress and those ≥13 meeting the criteria for serious or debilitating psychological distress. FDI = Functional Disability Inventory: no/minimal (0–12), mild (13–20), moderate (21–29), and severe (≥30). CD-RISC-10 = 10-item Connor–Davidson Resilience Scale (40), with higher scores indicating greater resilience. PCS-C = Pain Catastrophizing Scale for Children. ^ MCID defined as half the standard deviation of the mean baseline scores.

## Data Availability

The data presented in this study are available upon request from the corresponding author. The data are not publicly available due to patient privacy concerns.

## References

[1] Feldman D.E., Nahin R.L. (2021). National Estimates of Chronic Musculoskeletal Pain and Its Treatment in Children, Adolescents, and Young Adults in the United States: Data From the 2007–2015 National Ambulatory Medical Care Survey. J. Pediatr..

[2] Sherry D.D., Brake L., Tress J.L., Sherker J., Fash K., Ferry K., Weiss P.F. (2015). The Treatment of Juvenile Fibromyalgia with an Intensive Physical and Psychosocial Program. J. Pediatr..

[3] Tran S.T., Guite J.W., Pantaleao A., Pfeiffer M., Myer G.D., Sil S., Thomas S.M., Ting T.V., Williams S.E., Edelheit B. (2016). Preliminary outcomes of a cross-site cognitive-behavioral and neuromuscular integrative training intervention for juvenile fibromyalgia. Arthritis Care Res..

[4] Gmuca S., Sherry D.D. (2017). Fibromyalgia: Treating Pain in the Juvenile Patient. Paediatr. Drugs.

[5] Groenewald C.B., Wright D.R., Palermo T.M. (2015). Health care expenditures associated with pediatric pain-related conditions in the United States. Pain.

[6] Rosenberg A.R., Yi-Frazier J.P., Eaton L., Wharton C., Cochrane K., Pihoker C., Baker K.S., McCauley E. (2015). Promoting Resilience in Stress Management: A Pilot Study of a Novel Resilience-Promoting Intervention for Adolescents and Young Adults With Serious Illness. J. Pediatric Psychol..

[7] Winsett R.P., Stender S.R., Gower G., Burghen G.A. (2010). Adolescent self-efficacy and resilience in participants attending A diabetes camp. Pediatric Nurs..

[8] Rosenberg A.R., Ms M.C.B., McCauley E., Curtis J.R., Wolfe J., Baker K.S., Yi-Frazier J.P. (2018). Promoting resilience in adolescents and young adults with cancer: Results from the PRISM randomized controlled trial. Cancer.

[9] Rosenberg A.R., Bradford M.C., Barton K.S., Etsekson N., McCauley E., Curtis J.R., Wolfe J., Baker K.S., Yi-Frazier J.P. (2019). Hope and benefit finding: Results from the PRISM randomized controlled trial. Pediatr. Blood Cancer.

[10] Rosenberg A.R., Steiner J., Lau N., Fladeboe K., Toprak D., Gmuca S., O’Donnell M.B., Smith K., Brown C.E., Yi-Frazier J.P. (2021). From Theory to Patient Care: A Model for the Development, Adaptation, and Testing of Psychosocial Interventions for Patients With Serious Illness. J. Pain Symptom Manag..

[11] Gmuca S., Xiao R., Urquhart A., Weiss P.F., Gillham J.E., Ginsburg K.R., Sherry D.D., Gerber J.S. (2019). The Role of Patient and Parental Resilience in Adolescents with Chronic Musculoskeletal Pain. J. Pediatr..

[12] Forrest C.B., Bevans K.B., Pratiwadi R., Moon J., Teneralli R.E., Minton J.M., Tucker C.A. (2014). Development of the PROMIS (R) pediatric global health (PGH-7) measure. Qual. Life Res..

[13] Forrest C.B., Tucker C.A., Ravens-Sieberer U., Pratiwadi R., Moon J., Teneralli R.E., Becker B., Bevans K.B. (2016). Concurrent validity of the PROMIS(R) pediatric global health measure. Qual. Life Res..

[14] Connor K.M., Davidson J.R.T. (2003). Development of a new resilience scale: The Connor-Davidson Resilience Scale (CD-RISC). Depress. Anxiety.

[15] Walker L.S., Greene J.W. (1991). The Functional Disability Inventory: Measuring a Neglected Dimension of Child Health Status. J. Pediatr. Psychol..

[16] Kessler R.C., Barker P.R., Colpe L.J., Epstein J.F., Gfroerer J.C., Hiripi E., Howes M.J., Normand S.-L.T., Manderscheid R.W., Walters E.E. (2003). Screening for Serious Mental Illness in the General Population. Arch. Gen. Psychiatry.

[17] Crombez G., Bijttebier P., Eccleston C., Mascagni T., Mertens G., Goubert L., Verstraeten K. (2003). The child version of the pain catastrophizing scale (PCS-C): A preliminary validation. Pain.

[18] Kashikar-Zuck S., Carle A., Barnett K., Goldschneider K.R., Sherry D.D., Mara C.A., Cunningham N., Farrell J., Tress J., DeWitt E.M. (2016). Longitudinal evaluation of patient-reported outcomes measurement information systems measures in pediatric chronic pain. Pain.

[19] Weiner B.J., Lewis C.C., Stanick C., Powell B.J., Dorsey C.N., Clary A.S., Boynton M.H., Halko H. (2017). Psychometric assessment of three newly developed implementation outcome measures. Implement. Sci..

[20] Proctor E., Silmere H., Raghavan R., Hovmand P., Aarons G., Bunger A., Griffey R., Hensley M.J.A. (2011). Outcomes for implementation research: Conceptual distinctions, measurement challenges, and research agenda. Adm. Policy Ment. Health.

[21] Kolbe R.H., Burnett M.S. (1991). Content-Analysis Research: An Examination of Applications with Directives for Improving Research Reliability and Objectivity. J. Consum. Res..

[22] Kleinheksel A.J., Rockich-Winston N., Tawfik H., Wyatt T.R. (2020). Demystifying Content Analysis. Am. J. Pharm. Educ..

[23] Flowers S.R., Kashikar-Zuck S. (2011). Measures of juvenile fibromyalgia: Functional Disability Inventory (FDI), Modified Fibromyalgia Impact Questionnaire-Child Version (MFIQ-C), and Pediatric Quality of Life Inventory (PedsQL) 3.0 Rheumatology Module Pain and Hurt Scale. Arthritis Care Res..

[24] Mease P.J., Spaeth M., Clauw D.J., Arnold L.M., Bradley L., Russell I.J., Kajdasz D.K., Walker D.J., Chappell A.S. (2011). Estimation of minimum clinically important difference for pain in fibromyalgia. Arthritis Care Res..

[25] Toprak D., Nay L., McNamara S., Rosenberg A.R., Rosenfeld M., Yi-Frazier J.P. (2020). Resilience in adolescents and young adults with cystic fibrosis: A pilot feasibility study of the promoting resilience in stress management intervention. Pediatr. Pulmonol..

[26] Rosenberg A.R., Zhou C., Bradford M.C., Salsman J.M., Sexton K., O’Daffer A., Joyce P. (2021). Assessment of the Promoting Resilience in Stress Management Intervention for Adolescent and Young Adult Survivors of Cancer at 2 Years: Secondary Analysis of a Randomized Clinical Trial. JAMA Netw. Open.

[27] Wakefield E.O., Puhl R.M., Litt M.D., Zempsky W.T. (2021). If It Ever Really Hurts, I Try Not to Let Them Know: The Use of Concealment as a Coping Strategy Among Adolescents With Chronic Pain. Front. Psychol..

[28] O’Donnell M.B., Adhikari E.A., Rosenberg A.R., Yi-Frazier J.P. (2021). Promoting Resilience for Pediatric Health Care Workers in the Era of COVID-19: The PRISM at Work Program. J. Palliat. Med..

[29] Yi-Frazier J.P., O’Donnell M.B., Adhikari E.A., Zhou C., Bradford M.C., Garcia-Perez S., Shipman K.J., Hurtado S.E., Junkins C.C., O’Daffer A. (2022). Assessment of Resilience Training for Hospital Employees in the Era of COVID-19. JAMA Netw. Open.

